# Copycats in Pilot Aircraft-Assisted Suicides after the Germanwings Incident

**DOI:** 10.3390/ijerph15030491

**Published:** 2018-03-11

**Authors:** Tanja Laukkala, Alpo Vuorio, Robert Bor, Bruce Budowle, Pooshan Navathe, Eero Pukkala, Antti Sajantila

**Affiliations:** 1 Mehiläinen Kielotie Health Centre, Vantaa 01300, Finland; tanja.laukkala@kela.fi; 2 Department of Forensic Medicine, University of Helsinki and Mehiläinen Airport Health Centre, Lentäjäntie 1 E, 01530 Vantaa, Finland; 3 Royal Free Hospital, Pond Street, London NW3 2QG, UK; robertbor@hotmail.com; 4 Centre for Aviation Psychology, London NW3 1ND, UK; 5 Center for Human Identification, University of North Texas Health Science Center, 3500 Camp Bowie Blvd., Fort Worth, TX 76107, USA; bruce.budowle@unthsc.edu; 6 Center of Excellence in Genomic Medicine Research (CEGMR), King Abdulaziz University, Jeddah 21577, Saudi Arabia; 7 The Maitland Hospital, Maitland 2320, Australia; pooshan.navathe@hnehealth.nsw.gov.au; 8 Faculty of Social Sciences, University of Tampere, 33100 Tampere, Finland; eero.pukkala@cancer.fi; 9 Department of Forensic Medicine, University of Helsinki, 00014 Helsinki, Finland; Antti.sajantila@helsinki.fi

**Keywords:** aircraft-assisted pilot suicide, copycat phenomenon, Werther effect, aviation safety

## Abstract

Aircraft-assisted pilot suicide is a rare but serious phenomenon. The aim of this study was to evaluate changes in pilot aircraft-assisted suicide risks, i.e., a copycat effect, in the U.S. and Germany after the Germanwings 2015 incident in the French Alps. Aircraft-assisted pilot suicides were searched in the U.S. National Transportation Safety Board (NTSB) accident investigation database and in the German Bundestelle für Flugunfalluntersuchung (BFU) Reports of Investigation database five years before and two years after the deliberate crash of the Germanwings flight into the French Alps in 2015. The relative risk (RR) of the aircraft-assisted pilot suicides was calculated. Two years after the incident, three out of 454 (0.66%) fatal incidents were aircraft-assisted suicides compared with six out of 1292 (0.46%) in the prior five years in the NTSB database. There were no aircraft-assisted pilot suicides in the German database during the two years after or five years prior to the Germanwings crash. The relative aircraft-assisted pilot suicide risk for the U.S. was 1.4 (95% CI 0.3–4.2) which was not statistically significant. Six of the pilots who died by suicide had told someone of their suicidal intentions. We consider changes in the rate to be within a normal variation. Responsible media coverage of aircraft incidents is important due to the large amount of publicity that these events attract.

## 1. Introduction

Aircraft-assisted pilot suicide is an extremely rare event. However, the consequences may affect a large number of bystanders [1,2,3,4]. Regular aeromedical assessments are mandatory for pilots and several suicide risk factors, such as previous suicide attempts or acute major depressive disorder, limit or prevent fitness to fly [5,6]. Estimates of aircraft-assisted pilot suicides from the U.S. between 1993 and 2002 were 0.44% (16 of 3648 fatal accidents) [7] and between 2003 and 2012 were 0.22% (eight of 3596 fatal accidents), showing an overall 20-year period estimate of aircraft-assisted suicides in the United States (1993–2012) to be 0.33% (95% CI 0.21–0.49; 24/7244 cases) [8]. These results are similar to an in-depth analysis of aircraft-assisted pilot suicides during the 2003–2012 period [9]. In a German study, based on Bundestelle für Flugunfalluntersuchung (BFU) accident investigations were conducted from 1974 until 2007 [10]. Suspected or confirmed suicides by aircraft resulted in 18 fatalities from nine separate cases over a period of 34 years (0.3 suicides per year) [10]. Furthermore, in two of these cases, the pilot was shot or stabbed and was a victim of a homicide-suicide [10]. These studies included general aviation pilots as well as commercial pilots. A European study on 28,000 male cockpit crew members revealed a standardized mortality ratio (SMR) of 0.63 (95% CI 0.48–0.79) on completed suicides irrespective of method [11].

When a sensational event occurs, copycats, defined for the purpose of this study as suicidal behavior provoked by media exposure, may arise; this is also described as the “Werther effect” (from Goethe’s novel The Sorrows of Young Werther) [12]. The term “Papageno effect” has been introduced to describe protective media measures, and refers to a birdcatcher character from Mozart’s Magic Flute, who became suicidal but recovered after his friends intervened [12,13]. In the literature on the copycat phenomenon, follow-up times vary markedly from short-term responses within days or weeks to long-term responses within years of an event [14,15,16,17,18,19,20,21,22]. These differences limit the comparability of data between studies. The emergence of copycat suicides has repeatedly been described in reference to celebrity deaths (please see e.g., [16,22]) where the specific method of suicide is copied, for instance jumping from particular bridges, being hit by a train or charcoal burning suicide [12].

After the deliberate Germanwings incident in the French Alps on 24 March 2015, there has been concern over a possible copycat effect in aircraft-assisted pilot suicides [2,4,23,24,25]. The Germanwings incident shared some features with a fatal aviation crash in 2013 in Namibia [1,26]. The pilot of the Namibian flight had significant stressors such as a prolonged divorce process and the death of a son in a car accident, which was also a suspected suicide. The pilot was alone on the flight deck and subsequently caused the aircraft to collide with the terrain resulting in 33 lives being lost [26]. 

The aim of this study was to assess whether there is evidence of possible changes in the rates of pilot aircraft-assisted suicides in the U.S. or Germany after the March 2015 Germanwings incident in the French Alps. Due to the rarity of aircraft-assisted pilot suicides, both general aviation and commercial pilots were the focus of the study.

## 2. Materials and Methods 

The National Transportation Safety Board (NTSB) database was searched on 30 December 2017, using the following search words: “suicide”, “murder-suicide” and “homicide-suicide”. Only fatal aviation accidents in the U.S., with full formal accident investigation reports finalized at the time of the search and the cause of accidents assessed as pilot suicide in the accident investigations, were included as index cases in this study. The German Bundestelle für Flugunfalluntersuchung (BFU) investigation reports were searched 2 January 2018, for aircraft-assisted pilot suicides and only finalized reports on fatal aviation accidents were included. The relative risk (RR) of the aircraft-assisted pilot suicides in all fatal accidents was calculated to compare the two-year period after the accident in the French Alps (from 25 March 2015 to 24 March 2017) with 5 years before the accident (from 24 March 2010 to 23 March 2015) in the U.S. In addition, the NTSB accident investigations were analyzed to assess the descriptive factors and the clustering by time or geographic region in these events. 

## 3. Results

During the two years after the Germanwings incident in the French Alps, three out of 454 (0.66%) fatal accidents were classified as aircraft-assisted suicides compared with six out of 1292 (0.46%) five years prior to the incident in the U.S. The nine aircraft-assisted pilot suicide events are described in detail in Table 1. All deceased pilots were men with ages ranging from 22–62 years (mean 44 years). Only one passenger died in these incidents. The relationship of this passenger to the pilot was not reported in the accident investigation. Six of the pilots had told someone that they intended to kill themselves, five of them days before the incident, and two left suicide notes. One pilot had a diagnosed major depressive disorder. There were no aircraft-assisted pilot suicides in the finalized German Bundestelle für Flugunfalluntersuchung (BFU) investigation reports two years after or five years before the Germanwings accident. There was one fatal accident case during the two years after the Germanwings crash and 18 fatal accidents with 1–4 casualties per accident during the five-year time period before the Germanwings incident in the German database. Thus, the combined U.S. and German pilot aircraft-assisted suicides, based on accident investigation reports, would be 3/455 after the Germanwings incident and 6/1310 before the Germanwings incident.

In the postmortem toxicology analysis, available in detail from six of the U.S. pilot investigations, two analyses were positive for antidepressants (selective serotonin reuptake inhibitor citalopram) and one was positive for the benzodiazepine drug alprazolam. Ethanol was found in three postmortem toxicology analyses and in two cases it was likely ingested before the event, and in one case there was either ingestion or postmortem production. Recreational drugs were not observed in these incidents. All but one pilot had a valid medical certificate. The pilot who was flying without a medical certificate was reported to have lost it due to repeated alcohol use-related events. The manner of death was described as suicide for all pilots with available reported autopsy causes of death. Four of the pilots in this study were flight instructors. Details of the suicide notes were not available.

In one of the incidents, the pilot had a self-inflicted gunshot wound. In another fatal accident, classified as a suicide, the pilot had reported technical problems on the emergency frequency immediately prior to the event.

Event dates and geographical locations in the U.S. did not refer to clustering, but one of these suicides occurred within four weeks of the Germanwings crash (Table 1). The relative risk of the likelihood of an aircraft accident being due to pilot suicide after 24 March 2015, was 1.4-fold (95% CI 0.3–4.2) as compared with the likelihood during the five years before that.

## 4. Discussion

Approximately one out of 200 fatal aviation accidents in this study was pilot aircraft-assisted suicide. Accidents in commercial and airline aviation are reducing. Hence a comparison of the proportion of suicidal flight crashes out of all crashes would show some increase even if the proportion of suicidal crashes out of all flights would not increase. Moreover, due to the rarity of these events, the confidence interval of the relative risk estimate of 1.4 (0.3–4.2) allows for the decrease or increase of events but cannot confirm either. We consider the difference between the follow-up time periods of two and five years to be within normal random variation. Recently, even WHO [27,28] has paid attention to the need for long-term follow-up studies on the copycat phenomenon in order to further develop responsible reporting guidance to the media.

The copycat phenomenon has been regularly described in the literature [12,14,15,16,17,18,19,20]. Due to the rarity of aircraft-assisted pilot suicides, we chose to follow incidents for two years, which is a long-term follow-up period for copycats. Copycat suicides may cluster over time or place [29]. To understand the copycat phenomenon, social learning theory of copycat suicides assumes that there are already individuals experiencing thoughts of suicide in society and media impact can provoke them into action [19]. In a 1970s study, airplane accident fatalities increased after newspaper stories about murder and suicide [30].

Our review of the aircraft-assisted pilot suicides before and after the Germanwings accident did not show any evident clustering by geographic region or time in the U.S. This suggests that these are not copycat events. One accident occurred in the fourth week after the Germanwings accident. In this case, there were suicidal text messages, not accessible in the accident investigation material that might have clarified causes.

Identification to someone “special” is essential in copycat phenomenon, but it seems plausible that pilots would not identify themselves with the pilot of the ill-fated Germanwings event as media described it in various negative ways. Moreover, the pilot’s actions were associated with an immeasurable amount of grief to those affected. Homicide-suicide committers share many similarities to suicide committers, but the impact of negative life events may differ [2,4,31,32].

Social learning theory gives some guidance to responsible media coverage [19]. WHO [28] has emphasized the responsible media reporting of suicide prevention efforts. Reporting facts of accidents with information on how to obtain psychosocial support if needed (e.g., crisis hotlines), without emphasizing details provoking strong negative emotions, is considered responsible media coverage of suicides [28,33], but the need of guidance has also been questioned [34]. These principles seem to have been partly challenged in the reporting of the Germanwings accident [35] with the murder component being more strongly emphasized. There is, however, no evidence or suggestion that approaches to reporting incidents and events directly leads to or triggers suicidal behaviour. The guidance offered appears to be aimed at preserving the dignity and the sensitivity of these situations. Furthermore, murder-suicide is arguably a different event to suicide, with complex and more extensive consequences reaching into the lives of unsuspecting victims and their families who may not have had any suicide risk prior to the event.

Suicide rates fluctuate due to many causes in long-term follow-ups, which challenge efforts to assess the effectiveness of any evidence-based suicide prevention guidelines. Research on the Papageno effect and different forms of social media are an emerging area in suicidology, while there can be a possible bias of not reporting negative findings [12,36]. It should be noted that being involved with completed suicides and grieving of close family and friends can be stressful to all involved professionals including health care and psychosocial support professionals, accident investigators and other researchers and media professionals.

Suicidal intentions can be a sign of a psychiatric disorder. There was evidence of pilot psychiatric disorder or antidepressants detected in postmortem toxicology in 16/44 (36%) of general aviation pilot aircraft-assisted suicides in the study by Kenedi et al. [2]. In the study of the NTSB database for 51 aircraft-assisted pilot suicides over 32 years, all suicide committers were male, with an average age of 38 years, and 55% of these pilots had notified someone before the flight of their intentions [3]. Prescription medication (16% of the pilots), nonprescription medication (4%), and ethanol (14%) were present, suggesting the possibility of health-related issues by postmortem toxicology [3]. 

In this study, three out of nine individuals had psychotropic medications detected in the postmortem toxicology. Two pilots had antidepressants of which one was suffering from acute depression. In the general population suicide studies, the proportion of depression is generally higher. Selections to pilot schools favor physically and psychologically fit and resilient individuals, and several long-term psychiatric disorders are not compatible with flying duties [5,6,37,38]. The Aerospace Medical Association Working group [39] has suggested that more attention should be given to less serious and more common mental health issues and conditions during the aeromedical assessment of pilots. During the time period of this study, the treatment of major depression among pilots with flying duties may have changed, e.g., due to the development of a Human Intervention Motivation Study (HIMS)-trained Aviation Medical Examiner (AME) program and approved antidepressant use guidance. HIMS-AMEs may, in turn, evaluate pilots who wish to continue the use of SSRIs fluoxetine, sertraline, citalopram or escitalopram during the flying duties [5]. In clinical practice, recognition and treatment options for major depression have increased during the study period in the U.S., and as a consequence of updated European Aviation Safety Agency (EASA) regulations also in Europe. EASA efforts to provide rapid, unimpeded and pilot-centered psychological support and care should be encouraged in Europe.

According to the interpersonal theory of suicide, suicidal ideation is most likely to occur in the context of thwarted belongingness, perceived burdensomeness, reduced fear of suicide, and elevated physical pain tolerance [40]. Due to the nature of the job, commercial pilots often spend time irregularly separated from family. If such an individual has feelings of being a burden and alone, as well as unmet treatment needs, suicidal ideation could eventually develop [41]. The low standardized mortality ratio of 0.63 (95% CI 0.48–0.79) for suicide among male cockpit crew from nine European countries [11] supports the rarity of serious suicidal desire among generally resilient pilots. Their regular health examinations by AMEs are also a contributing issue, if all symptoms are truthfully reported.

Alcohol abuse has been reported in 14–26% of the aircraft-assisted pilot suicides [2,3]. Two out of nine pilots of this study were positive for ethanol in the postmortem analysis in amounts that might be considered alcohol abuse immediately before or during the flight accident.

Acute stressors related to private life appeared to affect over half of the pilots, which is comparable to the results of Kenedi et al. [2]. Negative life events and relationship issues may be even more influential in homicide-suicides [2,4]. Rice et al. [4] and Hussain et al. [42], among others, suggested lowering barriers of care as an effective non-stigmatizing suicide prevention intervention. This recommendation is in line with the International Civil Aviation Organization’s (ICAO) approach to evidence-based psychotherapeutic interventions generally in psychiatric symptoms [6]. Specific suicide prevention interventions were introduced earlier among aviators in the U.S. Air Force [43]. In this study, six of the pilots had told someone about their suicidal intentions, five of them within days of the aircraft-assisted suicide. While all suicides may not be preventable, implementation of high-quality evidence-based guidelines on suicide prevention could save lives [3,42,43,44,45].

Consideration also needs to be given to the implications of occupational de-licensing of professionals who may report or seek care for suicidal intent. This is a problem in aviation across all jurisdictions [46], and is also a problem in those jurisdictions where mandatory reporting of health professionals for such conditions is required [47].

Limitations of the present study include the fact that the manners of death assessments are based solely on data available from the accident investigations. Accident investigators may classify an event as suicide only when there is incontrovertible evidence, while all suicide completers do not leave suicide notes, for example [2,46]. Ongoing accident investigations might add relevant information. Data on aircraft-assisted pilot suicides are often limited due to the rarity of the phenomenon and limited information on the evaluation of possible psychiatric medical records. A psychological autopsy might be useful in the analysis of pilot suicides [3,8,23]. Data on the incidence of all pilot suicides irrespective of method, which was not accessible, would have been a valuable addition to this study. It is also important to consider that these pilots used aircrafts as a means of suicide. In relation to aircraft-assisted pilot homicide-suicides, other victims may hold different meanings from the pilot involved, while the means of suicide might have been copied and there is certainly individuality in these issues not related to profession.

## 5. Conclusions

There were six aircraft-assisted pilot suicides in the U.S. during the five-year period before the Germanwings accident and three aircraft-assisted pilot suicides during the two-year period after the Germanwings accident. Excluding the Germanwings accident, there were no aircraft-assisted pilot suicides during the study period in Germany according to the accident investigation database. Five of the nine U.S. pilots had conveyed their intentions within a few days before the accident. Due to very low numbers and arguably incomparable situations, there is no way to determine definitely the presence or absence of the copycat hypothesis. Training of the media in responsible news coverage may be considered to reduce the potential effects of media on suicides [27,28,45]. To assess the impact of the Germanwings incident in the general population, an investigation of all suicide methods, rates and timing would be needed. To clarify the possible serious harmful effects of the Germanwings incident among pilots, further research on fatal accidents with unclear causes would be warranted.

## Figures and Tables

**Table 1:** Aircraft-assisted suicides (all males) in the National Transportation Safety Board (NTSB) database five years before and two years after the Germanwings flight into the French Alps on 24 March 2015.

Event Date and State	Age (Years), Fatalities (*N*)	Medical Certificate **	Toxicology	Diagnoses	Cause of Accident (NTSB)	Other Information on Events before Incident Flight
**After the Germanwings Incident**						
12/15/2016 Alaska	62 *N* = 2	Class 3	None	No data	An act of suicide	No data
06/01/2016 Texas	45 *N* = 1	None (medical certificate denied)	Alprazolam butalbital metoprolol ethanol	No data	Intentional flight into a silo	Called his wife en route to airplane hangar. Told her he was going to kill himself
04/18/2015 California	34 *N* = 1	Class 1 *	Ethanol	No data	Intentional descent into terrain	Sent suicidal text messages to friends and family members earlier that day
**Before the Germanwings Incident**						
01/11/2015 Colorado	41 *N* = 1	Class 1 *	None	No data	Intentional descent into terrain	Pilot’s wife wanted a divorce. An earlier threat of flying airplane into the ground
07/22/2013 Virginia	22 *N* = 1	Class 1	Citalopram ethanol	Severe recurrent MDD	Intentional descent into ground	Fiancée contacted sheriff’s deputies because pilot intended to commit suicide. Suicide note
04/20/2013 Arizona	44 *N* = 1	Class 2	Reported done; no results given	No data	The pilot’s suicidal act	No data
09/23/2012 Atlantic Ocean, departed Florida	48 *N* = 1	Class 2 *	The body was not recovered	No data	The pilot’s suicidal act	Personal life difficulties. Joked before the accident about flying his plane into the ocean. Suicide note
03/11/2011 Arizona	47 *N* = 1	Class 2 *	Not performed	No data	Intentional flight into terrain	Relationship difficulties. Told a girlfriend on the day of the accident that he was going to kill himself
12/27/2010 Colorado	50 *N* = 1	Class 3	Citalopram	No data	Intentional flight into terrain	The pilot reported “losing elevator control” just before the accident

* Flight instructor, ** Class 1 = Airline Transport Pilot; 2 = Commercial Pilot; 3 = Private or Recreational Pilot, MDD = major depressive disorder.
